# Study on the High-Speed Milling Performance of High-Volume Fraction SiCp/Al Composites

**DOI:** 10.3390/ma14154143

**Published:** 2021-07-25

**Authors:** Youzheng Cui, Shenrou Gao, Fengjuan Wang, Qingming Hu, Cheng Xu, Fengxia Xu

**Affiliations:** 1 School of Mechanical and Electronic Engineering, Qiqihar University, Qiqihar 161006, China; gaoshenrou@163.com (S.G.); wfj320110@163.com (F.W.); huqingming1267@126.com (Q.H.); xucheng821226@163.com (C.X.); xufengxia_hit@163.com (F.X.); 2 Heilongjiang Province Collaborative Innovation Center for Intelligent Manufacturing Equipment Industrialization, Qiqihar 161006, China; 3 School of Mechatronics Engineering, Harbin Institute of Technology, Harbin 150001, China

**Keywords:** high-speed milling, SiCp/Al composites, surface quality, machining defects, cutting simulation

## Abstract

Compared with other materials, high-volume fraction aluminum-based silicon carbide composites (hereinafter referred to as SiCp/Al) have many advantages, including high strength, small change in the expansion coefficient due to temperature, high wear resistance, high corrosion resistance, high fatigue resistance, low density, good dimensional stability, and thermal conductivity. SiCp/Al composites have been widely used in aerospace, ordnance, transportation service, precision instruments, and in many other fields. In this study, the ABAQUS/explicit large-scale finite element analysis platform was used to simulate the milling process of SiCp/Al composites. By changing the parameters of the tool angle, milling depth, and milling speed, the influence of these parameters on the cutting force, cutting temperature, cutting stress, and cutting chips was studied. Optimization of the parameters was based on the above change rules to obtain the best processing combination of parameters. Then, the causes of surface machining defects, such as deep pits, shallow pits, and bulges, were simulated and discussed. Finally, the best cutting parameters obtained through simulation analysis was the tool rake angle *γ*_0_ = 5°, tool clearance angle *α*_0_ = 5°, corner radius *r* = 0.4 mm, milling depth *a_p_* = 50 mm, and milling speed *v_c_* = 300 m/min. The optimal combination of milling parameters provides a theoretical basis for subsequent cutting.

## 1. Introduction

The application of SiCp/Al composites in various fields has become increasingly more extensive. Because of its excellent performance, SiCp/Al composites are widely used in national defense and economic construction [1,2]. Due to the high hardness and high wear resistance of SiCp/Al composite materials, it becomes very difficult to control during the cutting process. During the machining process, handling SiCp/Al usually leads to severe deformation of the workpiece, increased tool wear compared to that of other materials, and higher processing costs. The low efficiency and poor surface processing quality of composite materials have greatly restricted the application of SiCp/Al [3,4], so it is urgent to solve the processing problem of this composite material. Using ABAQUS finite element software, Li et al. [5] established a two-dimensional simulation model of single particles, homogeneous materials, and multiple particles as well as the process of particle rupture in the processing of a large number of SiCp/Al composites under various processing parameters and investigated the temperature change. According to Xiang et al. [6], since the processing parameters are highly associated with the surface properties, a finite element simulation analysis of the processing of SiC particles was performed to investigate the effect of various ultrasonic amplitudes on the surface properties of SiCp/Al materials. A comparative study was carried out to confirm the accuracy of ULTM and existing machining theory and simulation. Based on the Johnson–Cook constitutive model and physical fracture criterion, Zhu et al. [7] established a thermo-mechanical coupled finite element model to evaluate the cutting process of 10% Al_2_O_3_/Al6061 composites with cemented carbide tools. The results show that as the feed rate increases, the cutting force increases, and the error between the cutting force and the measured value is only 9%. Pramanik et al. [8], set a volume fraction of 20% SiCp/Al composite material during cutting to monitor changes in the stress field and plastic deformation area. The interaction between the tool and particle was evaluated by finite element analysis. The simulation results show that the size and distribution of stress and strain, as well as the interaction between particles and tools, are the main reasons for particle fracture and delamination during cutting. Combining the advantages and ABAQUS/Specificity, Dandekar and Shin et al. [9] used a multi-step method to predict the surface damage of SiCp/Al materials with a volume ratio of 20%. In the two-stage hybrid finite element modeling method of Wang et al. [10], LS-DYNA software was used to establish a fast-cutting simulation model of SiCp/Al composite material with a volume fraction of 45%. The geometric characteristics of the machined surface and the types of surface defects were obtained with cutting experiments. Fathipour et al. [11,12] studied the two-dimensional cutting process of a SiCp/Al composite material with a volume fraction of 20% using ABAQUS/Explicit software and validated the model by comparing the shape of the chip with the experimental results. Shui et al. [13] developed a parameterized 2D-cutting simulation model of the volume integration rate and particle shape using the ABAQUS/explicit simulation software platform through the Python scripting language. Three types of finite element models were designed, such as a circle, regular polygon, and arbitrary polygon. By comparing the cutting forces predicted by the three models and the homogeneous metal model, the average cutting force of the composite was found to be approximately 20% higher than that of the homogeneous metal model.

Based on an analysis of the above literature, there are many studies on the cutting of low-volume fraction SiCp/Al materials nationally and abroad, but there are relatively few studies on the cutting of high-volume fraction SiCp/Al composite materials. In this experiment, using a cutting simulation of SiCp/Al materials, the composite material had an overall bonding coefficient of 65% and an average particle size of 30 μm (The average diameter of hard particles is 30 μm). Using the ABAQUS/explicit finite element simulation platform, the simplified micro-morphology model of 2D-multi-particle consistent hexagonal particles, containing the brittle fracture criterion, was established. The influence of cutting (temperature, force, stress) and chip was studied by changing the geometric angle and milling parameters, which provides a theoretical basis for future milling techniques.

## 2. Materials and Methods

### 2.1. Material Constitutive Model

To ensure the accuracy of subsequent finite element simulation results, it is particularly important to establish an accurate constitutive model of the matrix material. This article uses the Johnson–Cook material composition model that is commonly used in cutting simulations due to its robustness and efficiency [14,15,16,17]. Assuming that the flow stress of the model is primarily influenced by strain, the strain and temperature were calculated as follows:

(1) $$\overset{-}{\sigma }=\left[A+B{\left(\overset{-}{\varepsilon }\right)}^{n}\right]\left[1+C\ln\left(\frac{\overset{\cdot }{\overset{-}{\varepsilon }}}{{\overset{\cdot }{\overset{-}{\varepsilon }}}_{0}}\right)\right]\left[1-{\left(\frac{T-T_{room}}{T_{m}-T_{room}}\right)}^{m}\right]$$

where *A*, the yield strength of the material under greenhouse conditions; *n*, the strain-hardening coefficient; *m*, the thermal softening coefficient; *C*, the strain rate strengthening coefficient; $\overset{\cdot }{\overset{-}{\varepsilon }}$, equivalent plastic strain rate; $\overset{-}{\varepsilon }$, equivalent plastic strain; ${\overset{\cdot }{\overset{-}{\varepsilon }}}_{0}$, reference strain rate; *T_m_*, melting point temperature; *T_room_*, room temperature.

In the process of cutting simulation, the material to be cut is composed of Al matrix material and SiC reinforced particles. The average diameter of SiC particles in the SiCp/Al composite is 30 μm. The Johnson–Cook plastic parameters of the aluminum matrix material are shown in Table 1 and Table 2 and show the material property parameters of the numerical model.

### 2.2. Establishment of the Fracture Criterion Model of Aluminum Matrix Material

In this simulation analysis, the fracture failure criterion of the Al matrix material adopts the Johnson–Cook fracture criterion, which can better describe the whole process of fracture failure of the material under the condition of the geometric angle of the cutting thickness and the tool [18]. The Al matrix material failure parameters are shown in Table 3.

### 2.3. Establishment of the Fracture Criterion Model for SiC Particles

The brittle fracture of SiC particles mainly occurs during the SiCp/Al materials cutting process. Brittle fracture is divided into brittle shear and brittle fracture. Among them, brittle shear is reflected in the shear behavior after fracture, and brittle fracture is reflected in the fracture toughness of the material. The brittle fracture criterion describes the entire process of brittle fracture and the attenuation evolution of SiC particles [19,20].

This cutting simulation employs the $G_{f}^{I}$ brittle crack propagation mode, the initial standard and maximum principal stress standard for SiC particle fracture, and its formula, as follows:

(2) $$\max(\sigma_{1},\sigma_{2},\sigma_{3})=\sigma_{b}$$

where $\sigma_{b}$, the tensile strength of SiC. The process of material fracture progression is controlled by the fracture energy after brittle cracking.

After the SiC particles begin to crack, the evolution behavior of the failure is controlled by the cracking criterion. The crack displacement *u_n_*_0_ is calculated as follows:

(3) $$u_{n0}=2G_{f}^{I}/\sigma_{tu}^{I}$$

where $G_{f}^{I}$ is material type I fracture energy; $\;\sigma_{tu}^{I}$ is failure stress during the formation of mode I crack.

The influence of shear stress on the damage propagation of granular SiC was described by the relationship between the shear stress retention model and crack deformation [21]. The shear modulus G_s_ of the SiC particulate material at the fracture growth stage can be calculated as follows:

(4) $$G_{s}=\rho \left(\varepsilon_{nn}^{ck}\right)G$$

where *G* is the shear modulus before material failure;${\;\rho (\varepsilon }_{nn}^{ck})$. The formula used to calculate the shear retention of the material is as follows.

(5) $$\rho \varepsilon_{nn}^{ck}={\left(1-\frac{\varepsilon_{nn}^{ck}}{\varepsilon_{max}^{ck}}\right)}^{p}$$

where $\varepsilon_{nn}^{ck}$ is the cracking strain of granular SiC; *p* and $\varepsilon_{max}^{ck}$ are the material parameters. The relevant parameters required by the brittle fracture mode of the current material are shown in Table 4.

### 2.4. Establishment of the Friction Model

By comparing the maximum shear stress *τ_s_* and the friction shear stress *τ_f_* at different contact points, the magnitude of the two determines whether the node belongs to the bonded friction zone. This article uses the modified coulomb friction model, and the formula is as follows [22]:

When

(6) $$\tau_{f}\;\geq \;\tau_{s}\;(\mathrm{bonding}\;\mathrm{zone})\;\tau_{f}=\tau_{s}$$

When

(7) $$\tau_{f}\;<\;\tau_{s}\;(\mathrm{sliding}\;\mathrm{zone})\;\tau_{f}=\mu \sigma_{n}$$

where $\tau_{f}$, friction shear stress; $\tau_{s}$, maximum shear stress of the workpiece; $\sigma_{n}$, normal stress at this point, referring to [23]; *μ*, the friction factor, using 0.3.

## 3. Results and Discussion

### 3.1. Simulation Scheme Design

In the milling process, when different rake angles and corner radii are used for milling, the cutting force, cutting temperature, cutting stress, and chip will change accordingly. Because the single-factor variable method can clearly find the change trend and influence law of this variable when other factors remain unchanged, it is convenient for subsequent comparison and the optimization of tool parameters. When the machining depth was 0.2 mm and the milling speed was 300 m/min, the cutting simulation was carried out for different tool rake angles and tool tip arc radii through the parameter design method of a single-factor variable. Finally, by comparing the simulation results, the optimal parameters of different rake angles and corner radii were selected. Different tool parameter schemes are shown in Table 5 and Table 6.

Based on the follow-up simulation results, the optimal tool parameters are the tool clearance angle *α*_0_ = 5°, tool rake angle *γ*_0_ = 5°, and corner radius *r* = 0.4 mm. On this basis, the influence of different milling depths and milling speeds on the milling simulation results were studied by changing the milling parameters. By comparing the simulation results of cutting (force, temperature, stress) and chip, the corresponding laws were obtained, and the best machining scheme was selected according to the law. Different milling parameter schemes are shown in Table 7 and Table 8. 

### 3.2. Effect of the Tool Rake Angle on the Cutting Force

**Figure 1:**
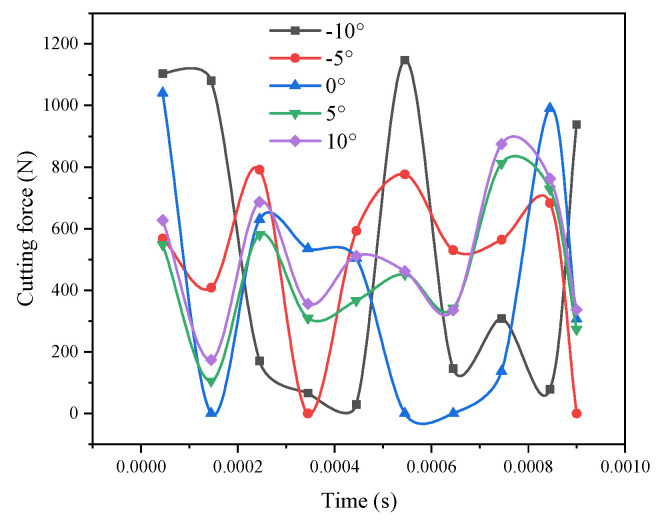
Comparison curve of the cutting force under different tool rake angles.

If you change the inclination angle of the tool, you can see that the inclination angle of the tool increases and the cutting force decreases significantly (Figure 1). This is because if the inclination angle of the tool is small, the contact area of the workpiece and the tool increases, which causes protrusion during the milling process. Moreover, SiCp/Al materials contain high-volume fraction SiC, so the properties of the material are close to brittleness, and anti-extrusion ability is relatively strong, so it will produce a large cutting force. However, when the tool rake reaches 10 degrees, the cutting force will gradually increase. This is because as the tool angle improves, the tool strength gradually reduces, thus resulting in severe wear of the tool during processing. As the tool tip becomes blunt, chip accretion will form on the front cutter surface, making the rake angle smaller, so the cutting force will increase instantaneously. Therefore, it can be seen from the above conclusion that *γ*_0_ = 5° is the best value for the rake angle tool.

### 3.3. Influence of the Corner Radius on the Cutting Force

**Figure 2:**
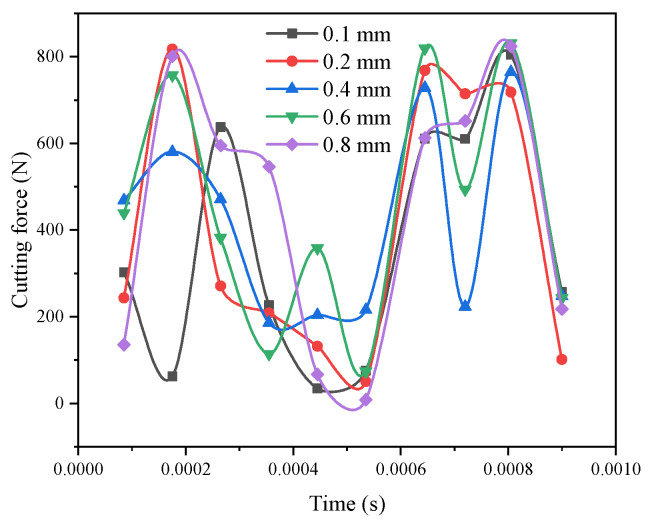
Comparison curve of the maximum cutting force under different corner radii.

Figure 2 compares the maximum cutting force at different corner radii. When the corner radius increases, the cutting resistance decreases, but when it reaches a certain value, the cutting resistance increases. This is because the gradual increase of the corner radius can appropriately reduce the tool wear and friction between the workpiece, but when the corner radius reaches a maximum peak, a relative vibration easily occurs between the workpiece and the tool, and the cutting force increases. At the same time, the excessive radius of corner radius can easily degrade the surface processing quality of the workpiece. From the above analysis, it can be concluded that the best parameter for the corner radius is *r* = 0.4 mm. 

### 3.4. The Effect of Milling Depth on the Cutting Force

**Figure 3:**
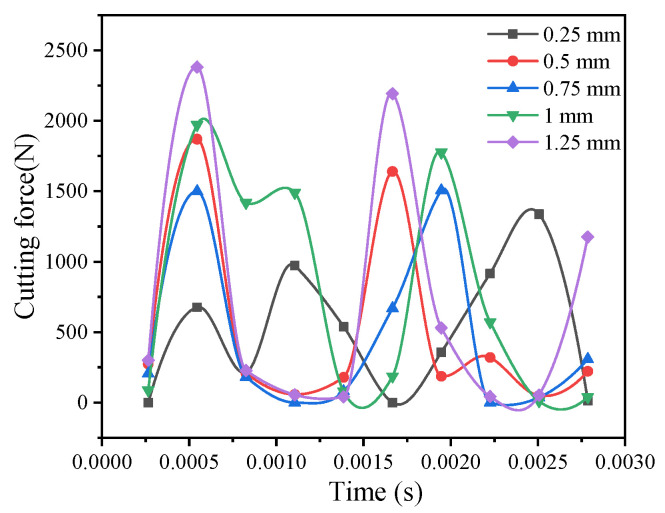
Cutting force curves at different milling depths.

Figure 3 shows the comparison curves of the maximum cutting force at various cutting depths. The cutting resistance improves as the machining depth improves, because the greater the machining depth, the greater the cutting resistance and the larger the cutting edge. On the contrary, the smaller the milling depth is, the smaller the cutting resistance and chip will be. At the same time, in the curve during the milling process, the cutting force instantaneously increases when contacting the tool SiC particles, which changes periodically.

### 3.5. Effect of the Milling Speed on the Cutting Force

**Figure 4:**
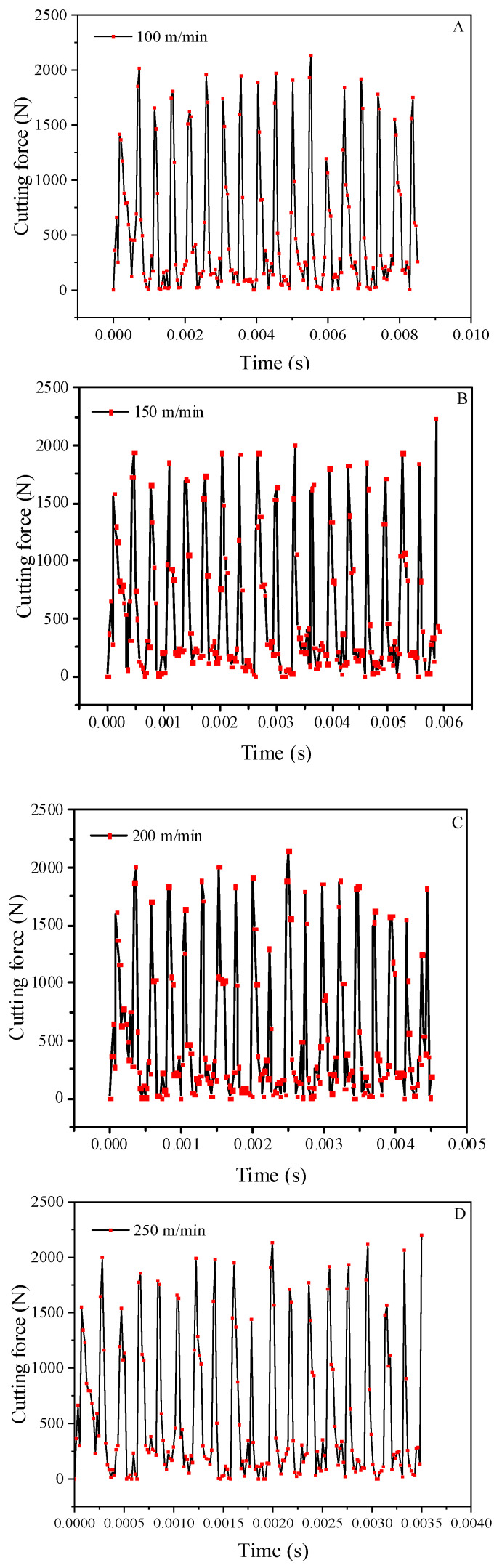

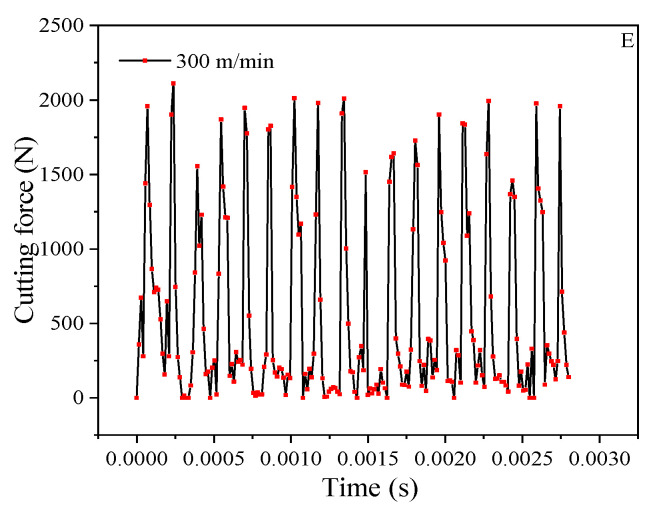
Curves of the cutting force at different milling speeds. (**A**) 100 m/min. (**B**) 150 m/min. (**C**) 200 m/min. (**D**) 250 m/min. (**E**) 300 m/min.

Figure 4 shows that as the processing speed improves, the cutting resistance increases slightly, but the trend of change is not obvious, so it is determined to be relatively stable. The reason is that the high-volume ratio of the SiCp/Al material is shallow, and the chips generated during the crushing process are not continuous, so the contact area between the workpiece and the tool is small, and thus the friction force is small. Therefore, there is no difference in the cutting force even when the speed is increased.

### 3.6. The Effect of the Tool Rake Angle on the Cutting Temperature

**Figure 5:**
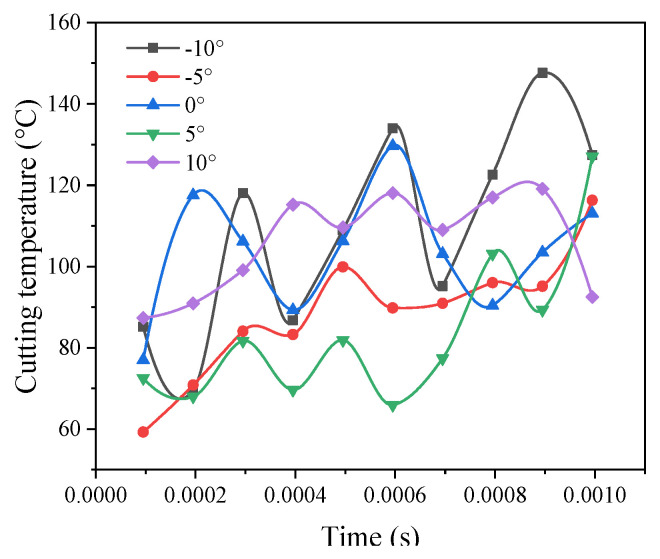
Comparison curve of the cutting temperature under different tool rake angles.

A comparison of the maximum cutting temperature under other tool inclination angles in Figure 5. As the tool inclination angle in the graph increases, the cutting temperature gradually decreases, but it can be seen from the figure that the angle *γ*_0_ = −5° results in a relatively low cutting temperature. The reason is that the tool is separated from the surface of the workpiece during the milling process, so the cutting temperature rises when the inclination angle *γ*_0_ = 10° is larger, as shown in the figure. The tool decreases and does not contribute to a decrease in temperature, so the temperature rises. From the comprehensive analysis of the above conclusions, the optimal value can be obtained when the tool rake angle is *γ*_0_ = 5°. 

### 3.7. Effect of the Corner Radius on the Cutting Temperature

**Figure 6:**
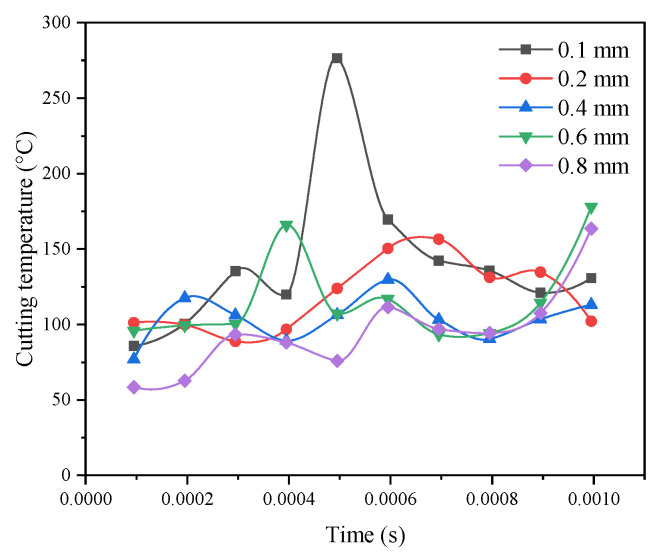
Comparison curve of the cutting temperature with different corner radii.

**Figure 7:**
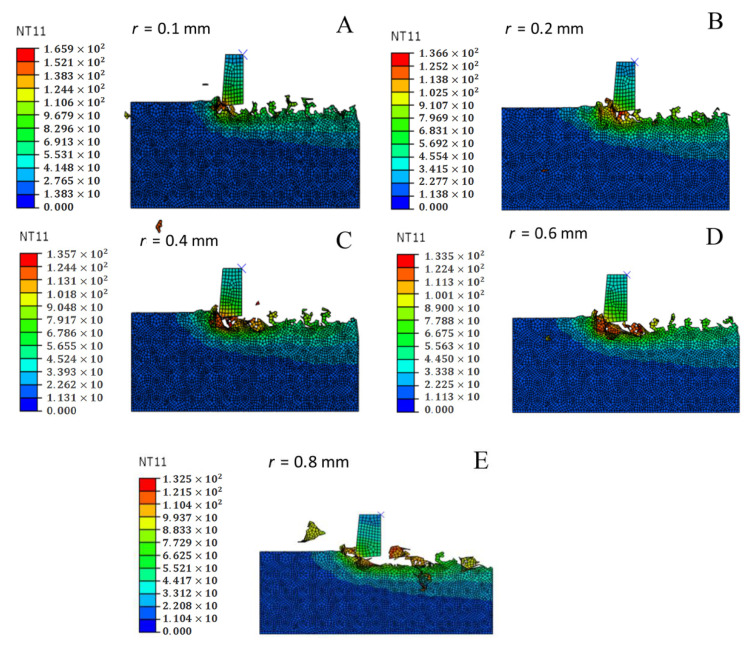
Cutting temperature nephogram with different corner radii. (**A**) *r* = 0.1 mm. (**B**) *r* = 0.2 mm. (**C**) *r* = 0.4 mm. (**D**) *r* = 0.6 mm. (**E**) *r* = 0.8 mm.

Figure 6 shows a comparison curve of the maximum cutting temperature when different corner radii were adopted and its effects on the milling process. The cutting temperature decreases as the corner diameter increases. The result can be confirmed by combining the cloud chart of the cutting temperature under different corner radii conditions, as shown in Figure 7. As the corner radius increases, the cutting temperature decreases. The reason for this phenomenon is that as the cutting chips increase and become discontinuous, the friction between the workpiece and the tool decreases, the friction heat decreases, and most of the cutting heat increases the cutting chips. Therefore, as the corner radius increases, the cutting temperature decreases. The optimal parameter can be obtained with the corner radius *r* = 0.4 mm, as determined through comprehensive analysis.

### 3.8. Effect of the Milling Depth on the Cutting Temperature

**Figure 8:**
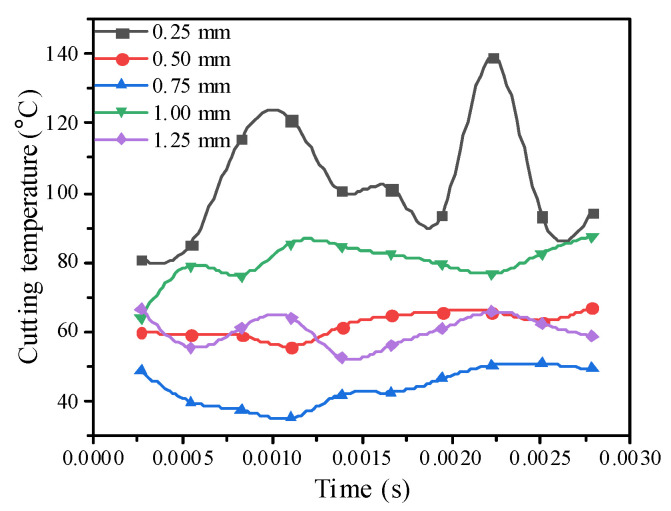
Comparison curve of the cutting temperature under different milling depths.

According to the comparison curve analysis of the maximum cutting temperature under different milling depths in Figure 8, wear can occur at any time, and the cutting temperature can be adjusted according to the gradually decreasing milling depth. However, when the milling depth is 0.75 mm, it is smaller because the tool tip does not contact the SiC particles with high hardness during the milling process, so the temperature is lower. The general rule is similar. As the milling depth increases, the cutting force of the tool to the workpiece gradually increases. and the generated cutting chip also becomes gradually larger and discontinuous. As a result, the friction between the workpiece and the tool is reduced, which directly reduced the cutting heat. Meanwhile, the increase of the chip also takes away most of the cutting heat, so, as the milling depth increases, the temperature changes, and as the depth of the cut improves, the temperature tends to decrease.

### 3.9. Influence of the Milling Speed on the Cutting Temperature

**Figure 9:**
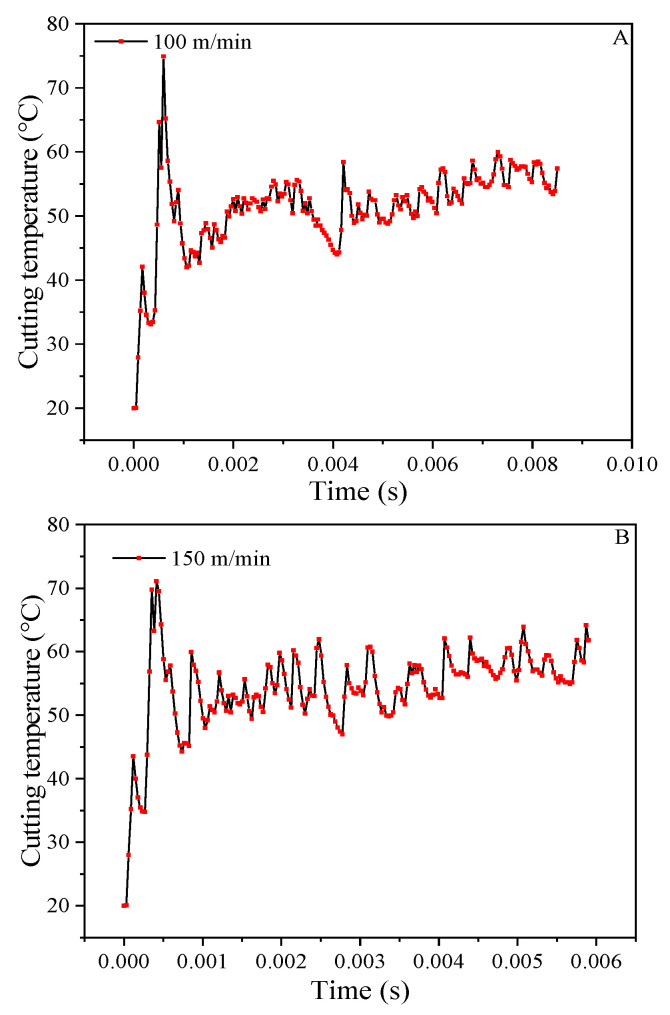

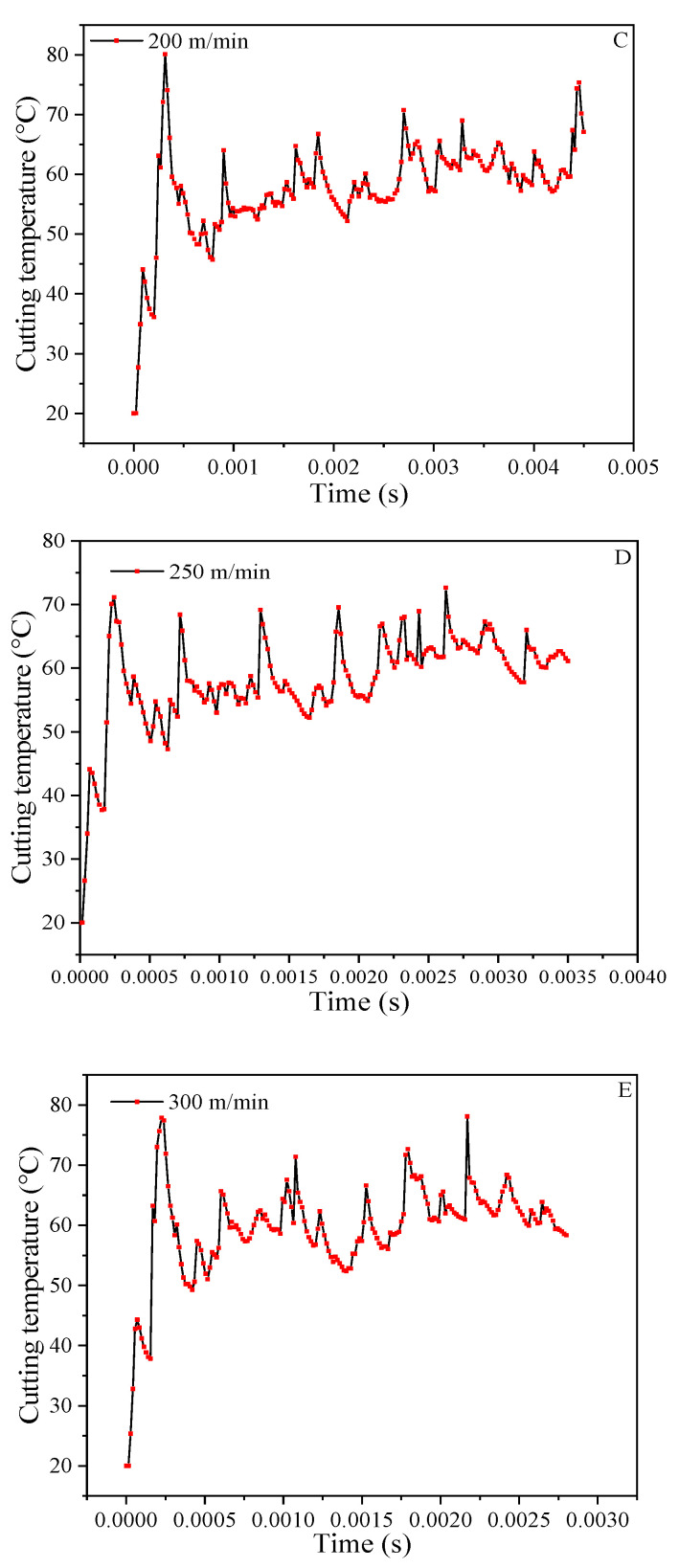
Cutting temperature curves at different milling speeds. (**A**) 100 m/min. (**B**) 150 m/min. (**C**) 200 m/min. (**D**) 250 m/min. (**E**) 300 m/min.

The change curve of the cutting temperature during the cutting process at the five processing speeds of 100 m/min to 300 m/min as shown in Figure 9. With five milling speed curves, as the milling speed improves, the temperature of cutting continues to rise and the fluctuations in temperature gradually stabilize. The reason is that as the milling speed improves, the impact and friction on the tool component also improves, and the temperature increases accordingly.

### 3.10. Effect of the Tool Rake Angle on Cutting Stress

**Figure 10:**
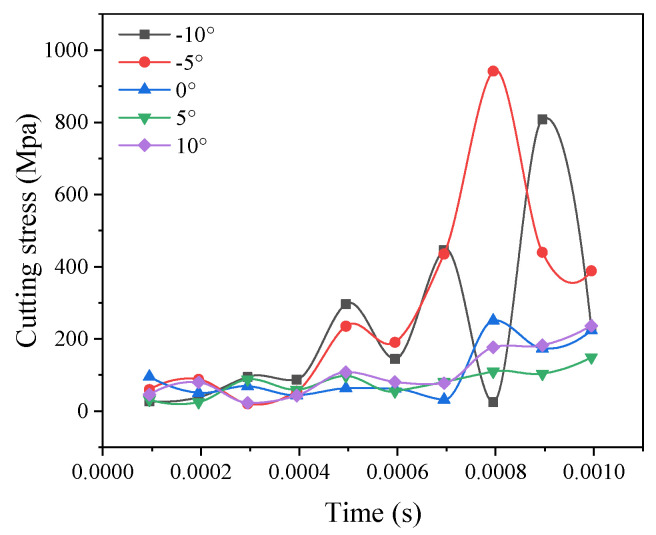
Comparison curve of the maximum stress of different tool rake angles.

As shown in Figure 10, cutting stress decreases and tends to stabilize with an increase in the inclination angle. As shown in Figure 10, the tool strength is reduced, and severely worn, with a blunt tool head, so that the tool tilt angle is not too large. Al-based materials easily accumulate on the slope to generate chips. The tools are mobilized, and the cutting stress increases. In addition, in the Figure 10, when the current angle is 5°, the cutting stress value is best. This further confirms the conclusion of the previous analysis.

### 3.11. Influence of the Corner Radius on Cutting Stress

**Figure 11:**
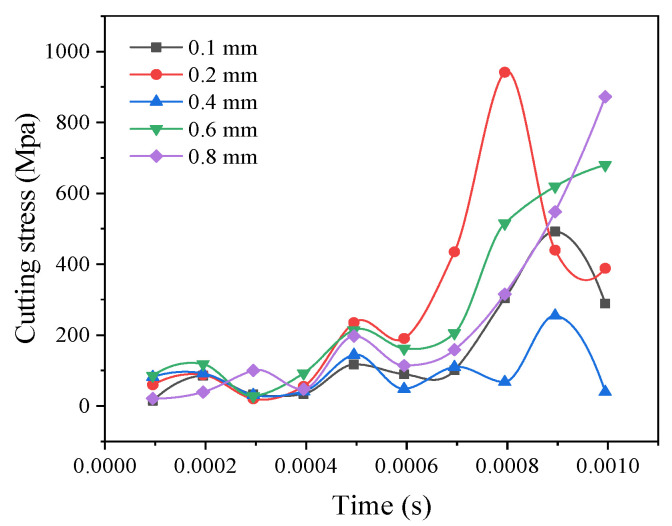
Comparison curve of the cutting stress with different corner radii.

As the corner radius improves, the cutting stress decreases (Figure 11), which is suitable when the corner radius is 0.4 mm. When the corner radius is 0.6 mm, the cutting stress begins to increase. This is because the corner radius increases, and the friction between the workpiece and the tool is small. The cutting stress will decrease, but when the corner radius continues to increase, there will be a mutual vibration between the tool and the workpiece that will lead to increased tool wear and cutting stress.

### 3.12. Influence of the Milling Depth on Cutting Stress

**Figure 12:**
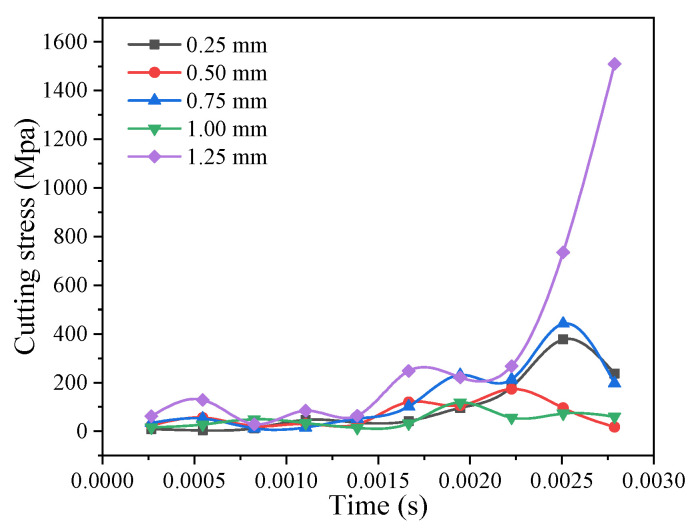
Comparison curve of the maximum cutting stress at different milling depths.

The maximum cutting stress at other machining depths is shown in the comparison curve in Figure 12. The cutting stress increases as the machining depth improves. As the depth of the milling machine increases, the contact area and friction between the workpiece and the tool increases, and the cutting resistance also improves. From the comparison curve, it can be determined that when the milling depth is 0.75 mm or 1.00 mm, the cutting stress had a downward trend, but it became larger when the milling depth was 0.25 mm. This is because the tool tip does not contact SiC particles during the milling process, so the cutting stress value will decrease; however, the overall change trend is basically the same. 

### 3.13. Influence of the Milling Speed on Cutting Stress

**Figure 13:**
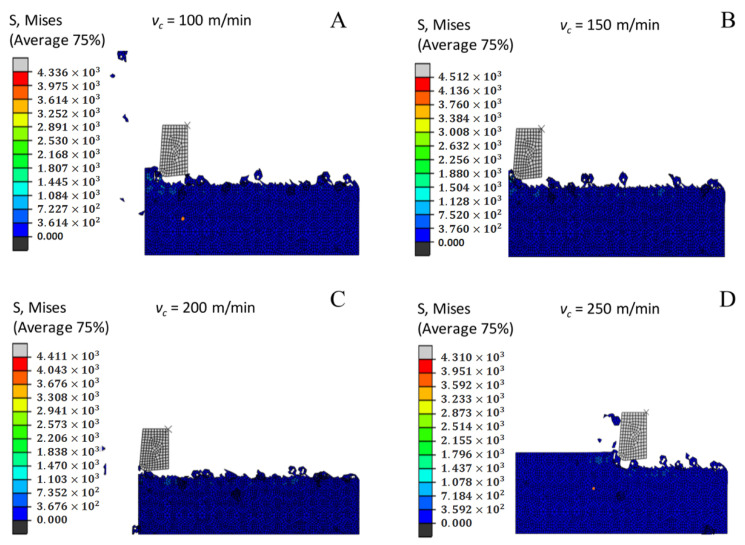

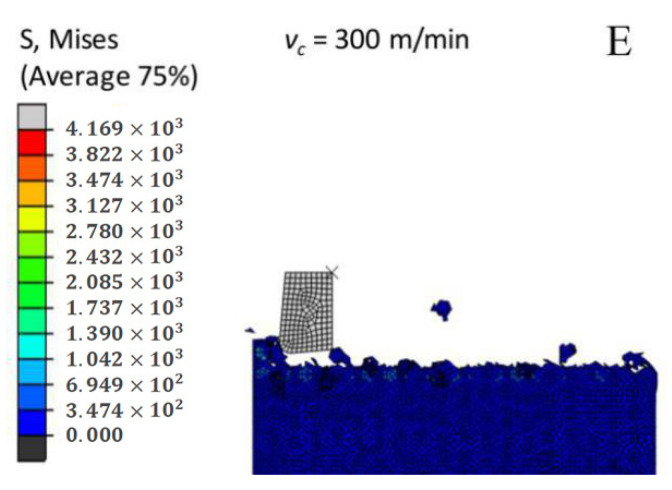
Cloud chart of cutting stress distribution under different milling speeds. (**A**) *v_c_* = 100 m/min. (**B**) *v_c_* = 150 m/min. (**C**) *v_c_* = 200 m/min. (**D**) *v_c_* = 250 m/min. (**E**) *v_c_* = 300 m/min.

Figure 13 shows a nephogram in which the cutting stress distribution at different milling speeds gradually decreases as the milling speed improves. The cutting speed increases and the base material is affected by the increased strain so that the connection of the chips and the flank of the tool that removes the chips from the base material without sufficient plastic deformation becomes insufficient. When the contacts are separated, the frictional resistance between the connection of the chips and the chip tool flank decreases, so the cutting stress decreases as the milling speed increases. 

### 3.14. The Effect of the Tool Rake Angle on Chip Formation and Morphology

**Figure 14:**
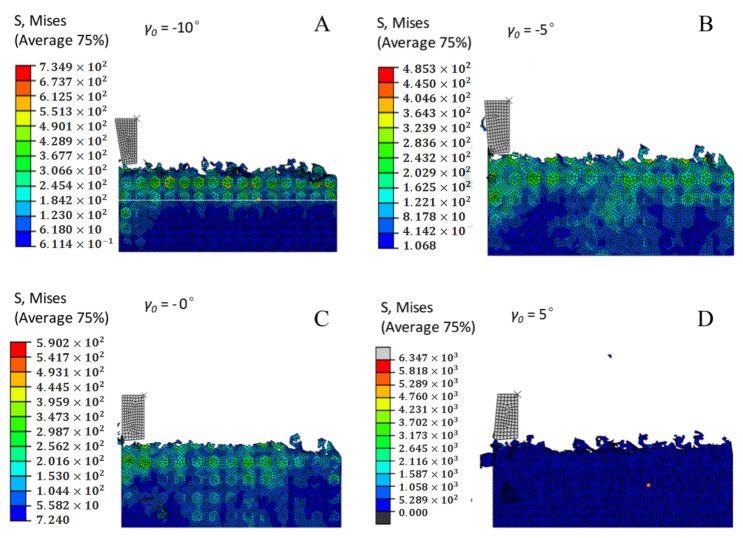

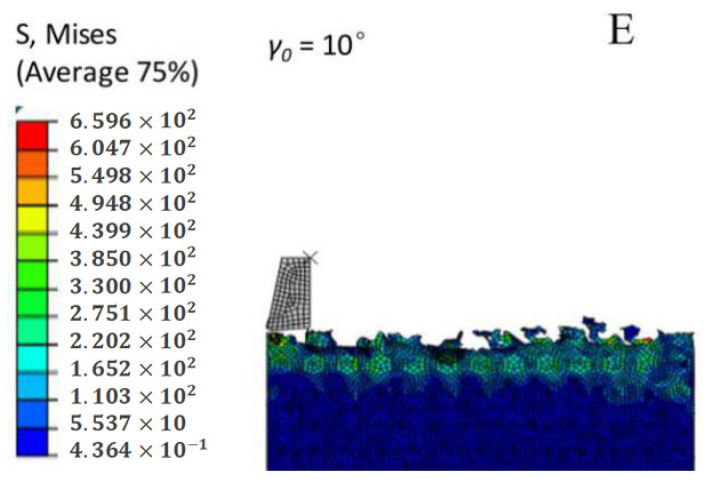
Chip morphology in milling with different tool rake angles. (**A**) *γ*_0_ = −10°. (**B**) *γ*_0_ = −5°. (**C**) *γ*_0_ = 0°. (**D**) *γ*_0_ = 5°. (**E**) *γ*_0_ = 10°.

Different tool rake angle pairs produce different chips in the process of cutting. Figure 14 shows the chip geometry obtained by milling at various tool rake angles. In the figure, you can see that the chip increases as the inclination angle of the tool increases. From Figure 14A,E, the electronic chip is much smaller in the former than in the latter. At the same time, the chip that formed is short, fragile, and discontinuous. The reason for this phenomenon is that SiC particles in SiCp/Al composites have high hardness, low plasticity, high brittleness, and easy deformation during cutting. At the same time, as the inclination angle of the tool increases, the tool becomes sharper and the plastic deformation of the chip is accelerated during the machining process, which is caused by comprehensive consideration.

### 3.15. Effect of the Corner Radius on Chip Formation and Morphology

**Figure 15:**
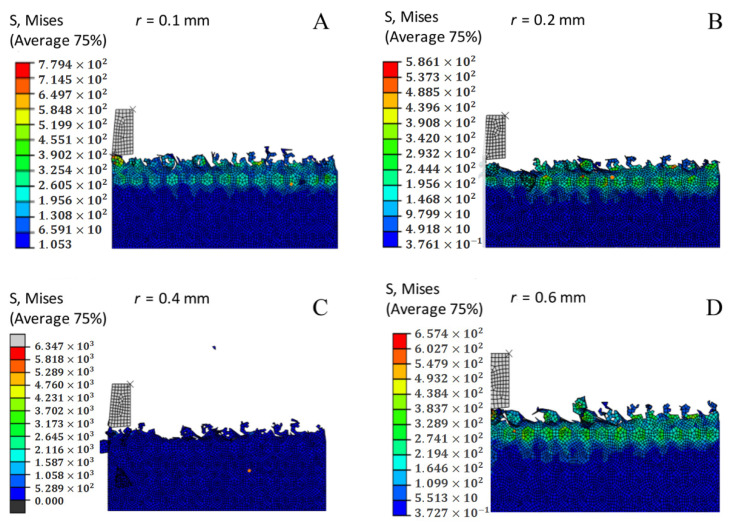

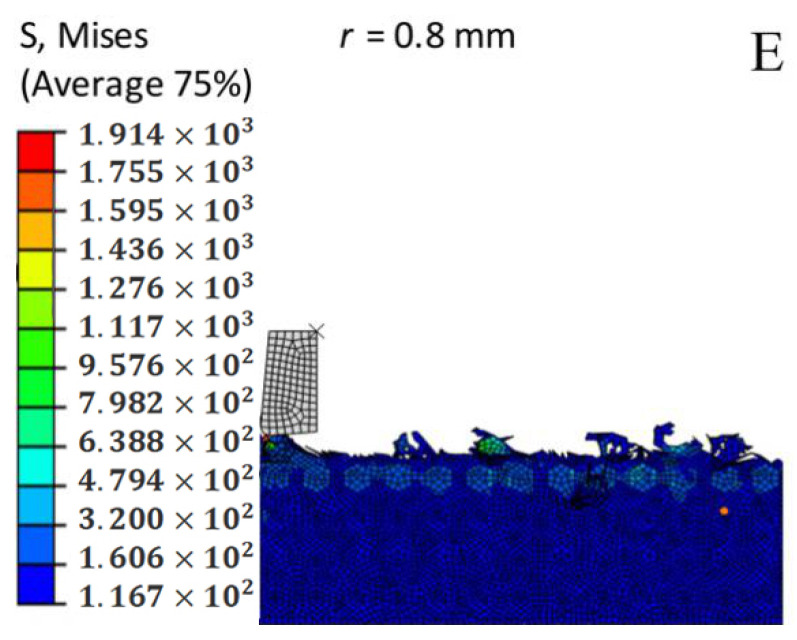
Chip morphology in milling with different corner radii. (**A**) *r* = 0.1 mm. (**B**) *r* = 0.2 mm. (**C**) *r* = 0.4 mm. (**D**) *r* = 0.6 mm. (**E**) *r* = 0.8 mm.

In the milling process, chip morphology will change as the corner radius changes. As can be seen by the chip shape in Figure 15, the larger the corner radius, the larger the chip size, and the larger the corner radius, the smaller the force exerted on the workpiece by the tool. At the same time, friction between the workpiece and the tool is also reduced. Due to the brittleness and hardness of the SiCp/Al composite, as the chip size increases the inch will increase with the increase of the corner radius.

### 3.16. Effect of the Milling Depth on Chip Formation and Morphology

**Figure 16:**
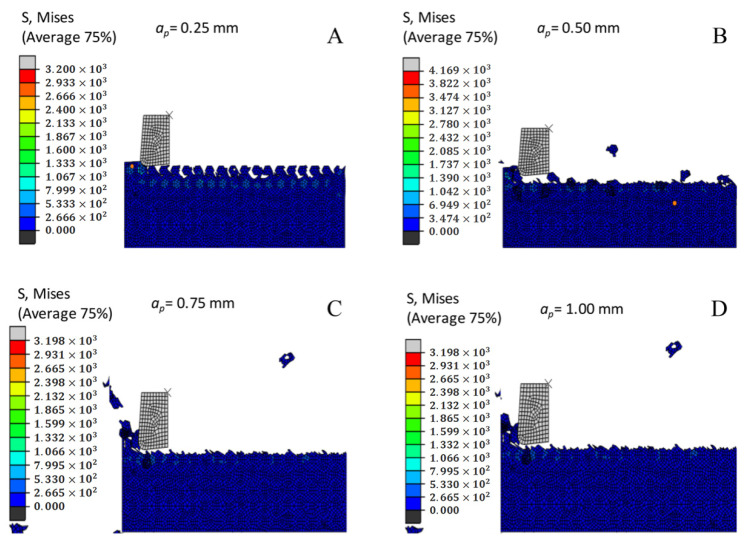

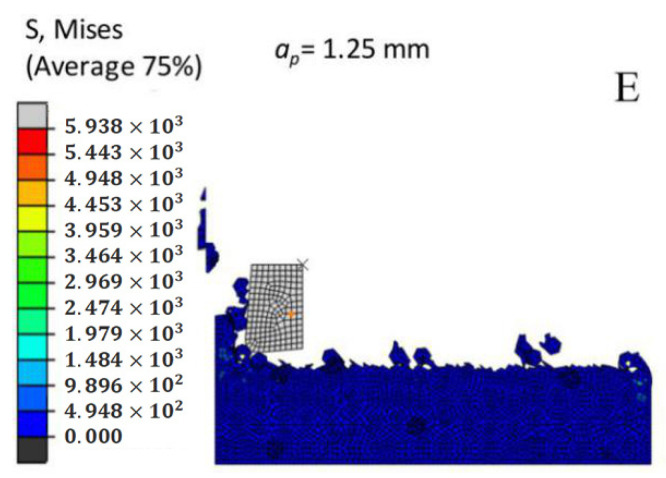
Chip morphology under different milling depths. (**A**) *a_p_* = 0.25 mm. (**B**) *a_p_* = 0.50 mm. (**C**) *a_p_* = 0.75 mm. (**D**) *a_p_* = 1.00 mm. (**E**) *a_p_* = 1.25 mm.

Milling depth is one of the key cutting parameters during the cutting process. It can be determined from the chip shape of other processing depth processes that as the processing depth increases, the chip size will increase; the shape of the chip is shown in the analysis conclusion. The reason is that as the depth of the milling cutter increases, the cutting resistance increases significantly, and the contact area between the cutter and the workpiece also improves. In the process of chip flow, the root of the chip is vulnerable to strong friction and extrusion of the rake face. Based on the material characteristics of the SiCp/Al composites, the cracks in the shear zone during the machining process are prone to sudden shear under the pressure of the tool, thus forming large and fragile chips (Figure 16).

### 3.17. Effect of the Milling Speed on Chip Formation and Morphology

**Figure 17:**
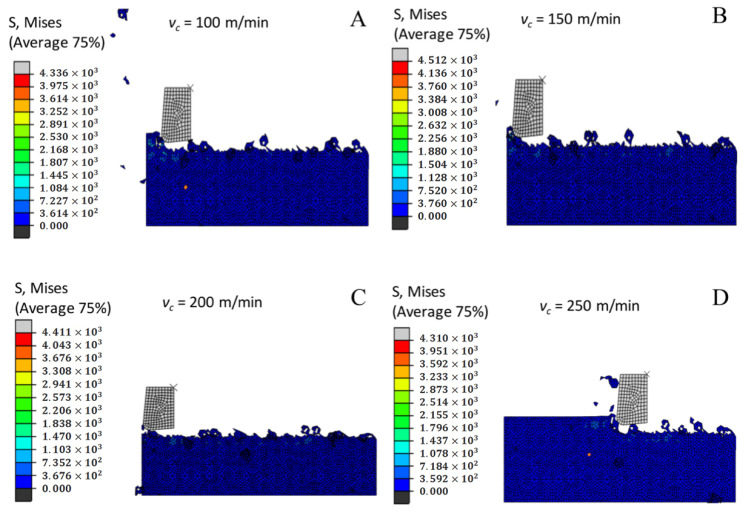

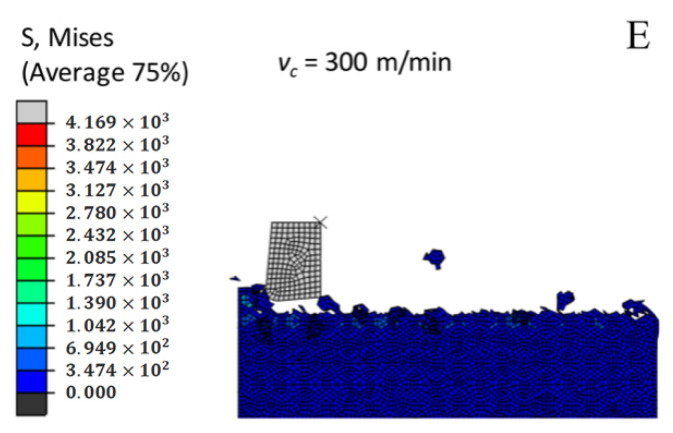
Chip morphology at different milling speeds. (**A**) *v_c_* = 100 m/min. (**B**) *v_c_* = 150 m/min. (**C**) *v_c_* = 200 m/min. (**D**) *v_c_* = 250 m/min. (**E**) *v_c_* = 300 m/min.

As can be determined by the chip shape of various milling speeds, as shown in Figure 17, the surface quality obtained in high-speed milling is much better than that of low-quality milling, and the chip size increases as the milling speed increases. The reason is that when the crushing speed is improved, the effect of the improved deformation rate of the metal matrix material on the cutting area is high, material flow is reduced, and accordingly, the retention of the SiC matrix material near the chip workpiece separation point improves. In high-speed milling, the rule of thumb is that the cutting temperature changes as the milling speed increases. The chip produced under a higher cutting temperature condition is easier to cut under the effects of high temperature, so the chip becomes larger and the substrate material easily peels away, thus improving the surface quality. Therefore, it is recommended to choose a high milling speed, as the processing parameter will ensure high surface quality.

### 3.18. Tool Selection

The polycrystalline diamond (PCD) tool used in this article has high wear resistance and should not produce a built-up edge during machining. Choosing a good tool parameter will have a profound impact by improving the milling efficiency and saving costs. As a result, the larger the tool inclination angle, the smaller the cutting resistance, cutting temperature, and cutting stress, and the larger the chip size. However, if the inclination angle of the tool is too large, the strength of the tool will greatly reduce, and the tool will become severely worn during processing, thus causing the tip of the tool to quickly become blunt. The inclination angle can be reduced by using the angle above the inclination, which can improve the cutting force and temperature instantly, thereby extending the life of the tool. The corner radius is an important parameter that determines the service life of the tool. According to the above comparison, as the corner radius increases, the cutting force, cutting temperature, and cutting stress gradually decrease, and the milling process improves. The larger the chips that are produced by the process, the better the surface quality of milling. Following research by some scholars [24], the tool wear can be appropriately decreased with a continuous improvement in the corner radius, but when the corner radius is too large, relative vibration is prone to occur between the workpiece and the tool. The surface processing accuracy of the workpiece is not high. At the same time, the milling depth and milling speed also have a great impact on the milling surface quality. Through the above simulation analysis, it was discovered that as the milling depth increases, the cutting force gradually increases and the cutting temperature gradually decreases, which changes the cutting stress and chip. These changes lead to larger and better surface finishes. As the milling speed improves, the cutting force increases but with little change, the cutting temperature gradually increases, and the cutting stress and chips gradually increase.

**Figure 18:**
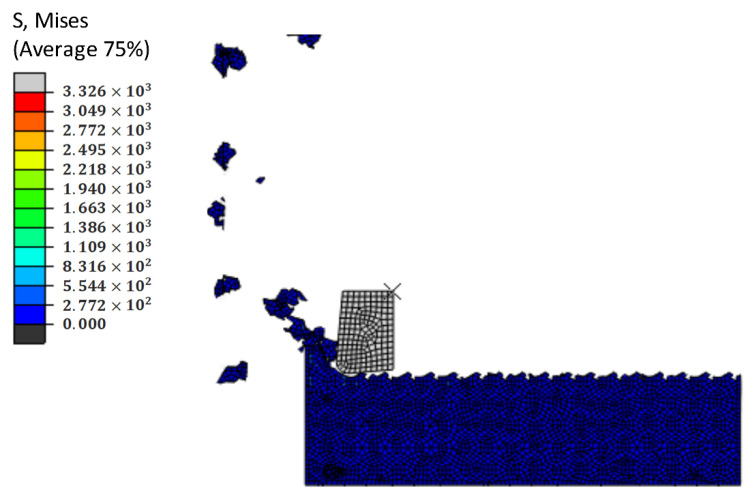
Surface topography under optimal cutting parameters.

So, in summary, considering the tool wear, service life, and surface processing quality, the simulation data is used to select the optimal tool processing combination of parameters, which are as follows: tool rake angle *γ*_0_ = 5°, corner radius *r* = 0.4 mm, milling depth *a_p_* = 1.00 mm, and milling speed *v_c_* = 300 m/min. As shown in Figure 18, the cutting topography of the optimal cutting parameters show the processing parameters that were demonstrated to achieve better surface processing.

### 3.19. Formation Mechanism and Process Analysis of the Shallow Pit

In processing SiCp/Al composites, adding SiC particles to the composites has brittle fracture characteristics. Therefore, surface defects such as shallow feet, deep feet, and protrusions are easily formed on the processed surface of the workpiece. Therefore, it is very practical to explore the formation process and causes of the above-mentioned surface quality defects with the aim to improve the surface treatment quality of SiCp/Al materials.

**Figure 19:**
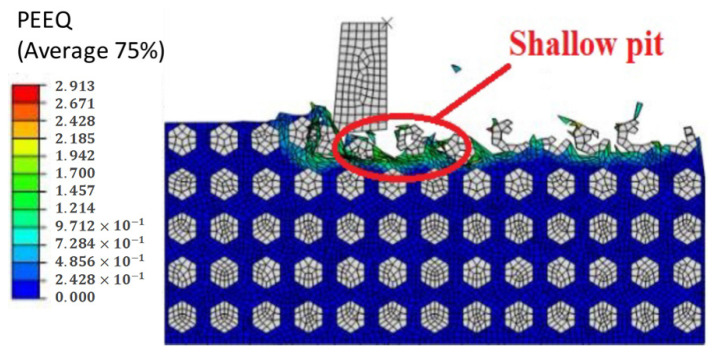
Simulation results of shallow pits on a machined surface.

**Figure 20:**
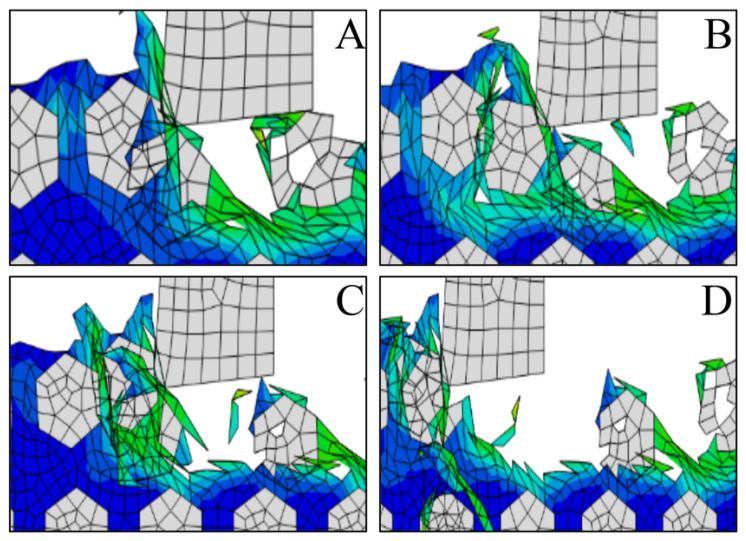
(**A**–**D**) Schematic diagram of the shallow pit formation process.

The distribution of SiC particles is shown in Figure 19. With the continuous simulation of cutting process, because the binding force of the Al matrix material on SiC particles is relatively small, the particles are pushed along the cutting direction, and the SiC particles start to flip continuously under the action of the tool, which leads to the particles separating from the matrix material and forming shallow pits. Figure 20 shows the whole process of shallow pit formation.

**Figure 21:**
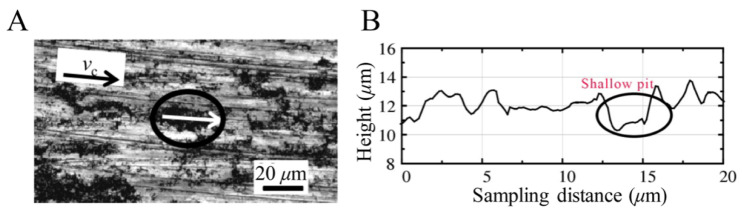
Typical surface shallow pit morphology [25]. (**A**) Surface sampling location. (**B**) Two-dimensional morphology.

As shown in Figure 21, the accuracy of the simulation results was verified by comparing the results of the existing processing experiments, the surface morphology of the SiCp/Al composite was observed with a laser confocal microscope after milling was complete, and the two-dimensional micro-morphology along the milling speed direction was extracted. As shown in Figure 21A, there is a typical shallow pit on the surface, and in Figure 21B is the two-dimensional morphology of milling. There is a shallow pit with a depth of 2 µm on the surface. It can be inferred that the reason for the formation of the shallow pit is that the particles are pulled out due to the mutual extrusion between the particles, so the existence of the shallow pit is further verified [25].

### 3.20. Formation Mechanism and Process Analysis of the Deep Pit

**Figure 22:**
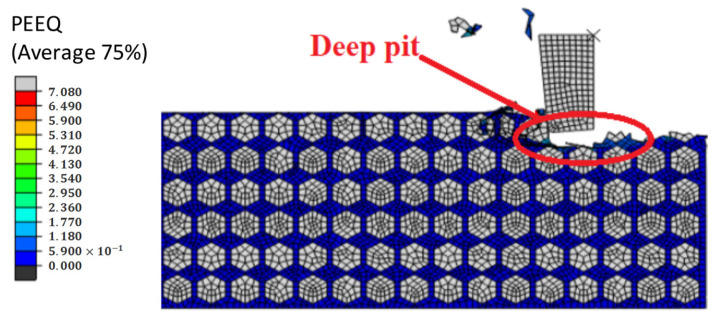
Simulation results of a deep pit on a machined surface.

**Figure 23:**
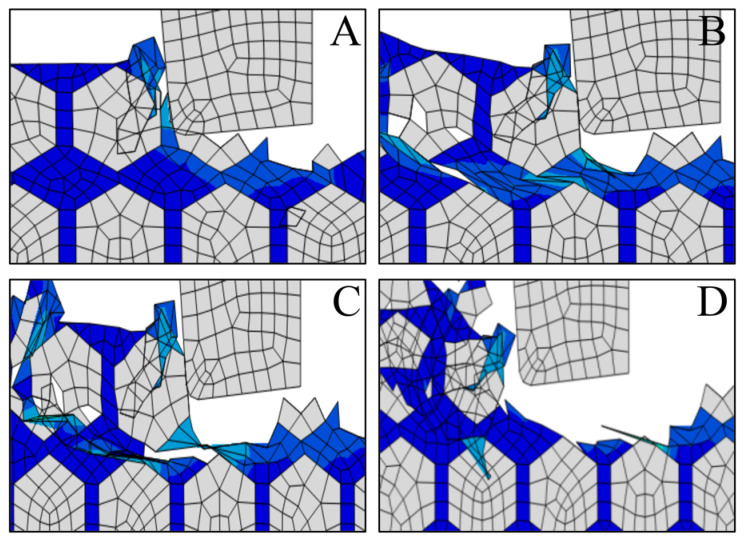
(**A**–**D**) Schematic diagram of the formation process of a deep pit.

Figure 22 shows the result of deep pits on the machined surface of the SiCp/Al materials. To further study the formation of deep pits, Figure 23 shows a schematic diagram of pits obtained by intercepting four different pit shapes at different stages. When the tool contacts the SiC particles after putting the notch in the workpiece, the SiC particles move in the direction of the grinding speed by action of the tool, and the area affected by the cutting part gradually expands, thus forcibly moving other parts to generate particles. It forms deep holes into the surrounding material.

**Figure 24:**
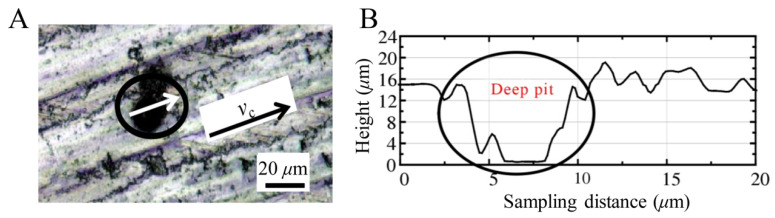
Typical surface deep pit morphology [25]. (**A**) Surface sampling location. (**B**) Two-dimensional morphology.

To verify the accuracy of the simulation results, the results of the conventional processing experiments were compared, as shown in Figure 24, and the surface micro-morphology of the SiCp/Al composite after milling was observed by a laser confocal microscope, and the two-dimensional morphology along the milling speed direction was extracted. The arrow marked in the black circle in Figure 24A represents the sampling position of the two-dimensional topography. It can be seen from Figure 24B that there is a deep pit with a depth of approximately 16 μm in the two-dimensional topography. It can be assumed that the deep pit formed mainly due to the interaction between the particles and the mutual protrusion and between the workpiece and the tool in the milling process. The results show that deep holes formed during the milling simulation process.

**Figure 25:**
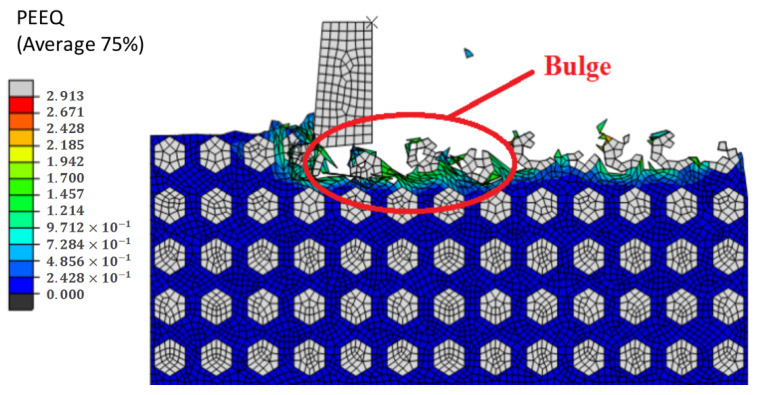
Simulation results of a machined surface bulge.

**Figure 26:**
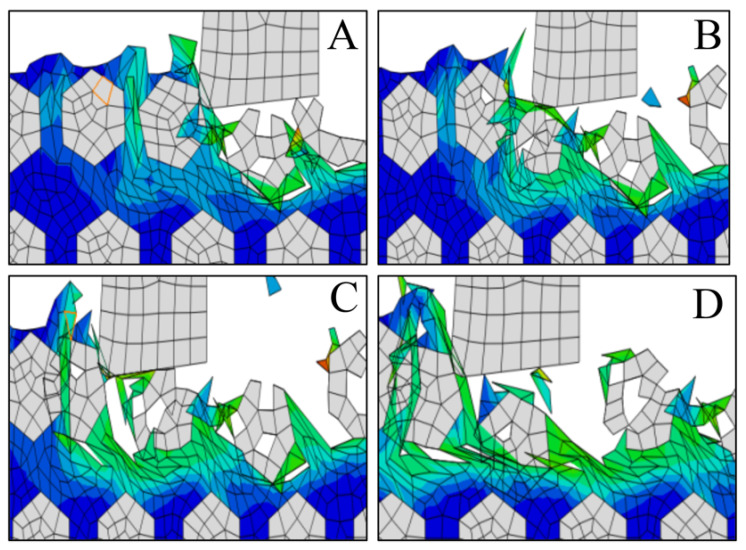
(**A**–**D**) Formation process of a machined surface bulge.

Simulation results of the formation obtained during processing can be viewed in Figure 25. As you can see, the protrusions appear because the particles did not completely fall off during the cutting process, and no particle tool formed in the protrusions. Figure 26 shows the process of protrusion formation. Under the action of the tool, the particles interact with each other, and after moving and overturning, the particles finally leave the workpiece, are exposed to the machined surface, and reach higher than other horizontal heights until they finally form a bulge.

**Figure 27:**
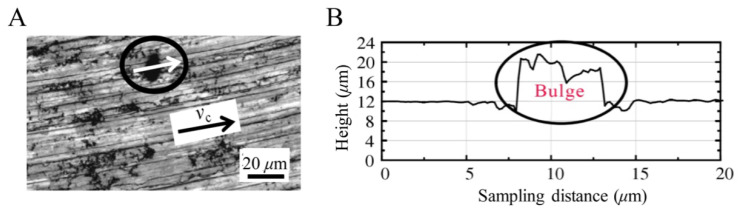
Typical surface bulge [25]. (**A**) Surface sampling location. (**B**) Two-dimensional morphology.

As shown in Figure 27, the accuracy of the simulation results is verified by comparing the results of the conventional processing experiments. The surface micro-morphology of the SiCp/Al composite after milling was taken by laser confocal microscope, and the two-dimensional morphology along the milling speed direction was extracted. Figure 27A shows the sampling position of the convex surface, and Figure 27B shows the real two-dimensional topography extracted from the milling test. It can be seen from the figure that a 12 μm bulge formed at the sampling position of the surface, which verifies the results that were obtained during the milling simulation.

## 4. Conclusions

This paper uses the ABAQUS/explicit large-scale finite element analysis platform to improve the processing of SiCp/Al materials. The optimized combination of processing parameters provides a theoretical basis for subsequent cutting techniques.

In this paper, ABAQUS/explicit large-scale finite element analysis platform is used to simulate the milling process of SiCp/Al composites, and the following main conclusions are obtained.

(1) The influence and change rule of cutting force, cutting temperature, cutting stress, chip shape in different machining conditions and tool parameters of SiCp/Al composite in milling process are obtained.

(2) Through comprehensive analysis, it is found that the larger the rake angle, the larger the radius of the tool tip, the smaller the milling depth and the greater the milling speed, the better the surface machining quality of the workpiece. Meanwhile, the tool rake angle *γ*_0_ = 5°, tool clearance angle *α*_0_ = 5°, the corner radius *r* = 0.4 mm, milling depth *a_p_* = 50 mm, milling depth *a_p_* = 0.50 mm and milling speed *v_c_* = 300 m/min are selected according to the service life of the cutter, they are the optimal tool parameters and provide theoretical basis for practical milling.

(3) Through the analysis of the milling surface, the formation process and causes of shallow pit, deep pit and bulge are obtained, which provides theoretical basis for proving the authenticity of the surface processing defects mentioned above.

## Figures and Tables

**Table 1:** Coefficient of the Johnson–Cook constitutive model for the Al matrix material.

*A* (MPa)	*B* (MPa)	*n*	*C*	*m*	${\overset{\cdot }{\overset{-}{\varepsilon }}}_{0}\left(ls\right)$	*T_melt_* (°C)	*T_room_* (°C)
224	165	0.375	0.006	0.877	0.0001	630	20

**Table 2:** Material property parameters.

Material	SiC	Al
Density ρ (kg/m^3^)	2960	2700
Elastic modulus E (GPa)	180	68.9
Poisson’s ratio	0.23	0.33
Thermal conductivity λ (W/m·K)	160	193
Coefficient of expansion α (1/K)	8.6 × 10^−6^	21.8 × 10^−6^
Specific heat capacity$\;C_{p}\;$(J/Kg·K)	0.75	900

**Table 3:** The failure parameters of the Johnson–Cook fracture criterion for Al matrix materials.

d1	d2	d3	d4	d5
0.13	0.13	−1.5	0.011	0

**Table 4:** SiC brittle fracture parameters.

Tensile Strength $\;\sigma_{b}\;$(MPa)	Material Type I Fracture Energy *G_I_* (J/m^2^)	Retention Factor *p*	Cracking Strain $\;\xi_{k1}$	Crack Opening Strain $\;\xi_{max}$
1500	30	1	0.0001	0.001

**Table 5:** Single-factor analysis plan for different tool rake angles.

No.	Parameter Name				
	Tool Rake Angle *γ*_0_ (°)	Tool Clearance Angle *α*_0_ (°)	Corner Radius *r* (mm)	Milling Speed *v_c_* (m/min)	Milling Depth *a_p_* (mm)
1	−10	5	0.4	300	0.2
2	−5				
3	0				
4	5				
5	10				

**Table 6:** Single-factor analysis scheme of different corner radii.

No.	Parameter Name				
	Tool Rake Angle *γ*_0_ (°)	Tool Clearance Angle *α*_0_ (°)	Corner Radius *r* (mm)	Milling Speed *v_c_* (m/min)	Milling Depth *a_p_* (mm)
1	5	5	0.1	300	0.2
2			0.2		
3			0.4		
4			0.6		
5			0.8		

**Table 7:** Single-factor analysis scheme for different milling depths.

No.	Parameter Name				
	Tool Rake Angle *γ*_0_ (°)	Tool Clearance Angle *α*_0_ (°)	Corner Radius *r* (mm)	Milling Speed *v_c_* (m/min)	Milling Depth *a_p_* (mm)
1	5	5	0.4	300	0.25
2					0.50
3					0.75
4					1.00
5					1.25

**Table 8:** Single-factor analysis scheme for different milling speeds.

No.	Parameter Name				
	Tool Rake Angle *γ*_0_ (°)	Tool Clearance Angle *α*_0_ (°)	Corner Radius *r* (mm)	Milling Speed *v_c_* (m/min)	Milling Depth *a_p_* (mm)
1	5	5	0.4	100	0.50
2				150	
3				200	
4				250	
5				300	

## Data Availability

All data included in this study are available upon request by contact with the corresponding author.
